# Awareness, Perceptions, and Desirability of Epidural Analgesia Among Pregnant Women in King Fahad University Hospital, Al Khobar

**DOI:** 10.7759/cureus.20146

**Published:** 2021-12-03

**Authors:** Eman S Al Sulmi, Manar M Al Yousef, Jenan A Almuslim, Rawan A Al Muslim, Zahra A Amailo, Fatimah A Alabbad

**Affiliations:** 1 Obstetrics and Gynecology, King Fahad University Hospital, Al Khobar, SAU; 2 Medicine, Imam Abdulrahman Bin Faisal University, Dammam, SAU

**Keywords:** perception, labor, epidural, awareness, analgesia

## Abstract

Background: Childbirth is a normal life event that is considered to have the most severe pain faced by women in their lifetime. However, this pain can be managed in many ways such as using epidural analgesia (EDA).

Objective: This study focuses on assessing the awareness, perceptions, and desirability of EDA among pregnant women at King Fahad University Hospital (KFUH), Al Khobar.

Methods: A cross-sectional study was conducted at KFUH in Al Khobar, Eastern Province of Saudi Arabia. The study took place from November to December 2020 by using a pre-designed questionnaire developed by the researchers based on a thorough literature review of prior studies. A total of 209 pregnant females participated in the study. The study included all pregnant women attending antenatal clinics and the emergency room at KFUH and we excluded postpartum women from the study.

Results: A total of 209 pregnant women participated in this two-month study and completed the questionnaire. The majority of women were between the age of 30-35 years old (33%), while a few (16%) were less than 24 years. The patient’s perception was found to be significantly influenced by age and household income. On the other hand, awareness of EDA complications and desirability was seen to be associated with income.

Conclusion: Study results demonstrate that the majority of pregnant women were aware, as well as had a positive perception and desirability toward using EDA for labor pain. On the other hand, pregnant women who had a lack of awareness and perception toward EDA may suffer more from labor pain, which will negatively affect their birth experience and may make it less enjoyable. Therefore, education about different types of analgesia, especially EDA, should be included in an antenatal care program.

## Introduction

Childbirth is a normal life event that is considered to have the most severe pain faced by women in their lifetime. This pain is manageable and can be influenced by medications and many other factors including age and psychological factors such as anxiety, emotional support, educational level, culture, and ethnicity [1]. Epidural analgesia (EDA) is considered an effective measure for labor pain relief if administered timely [2]. EDA allows women to rest if the labor is prolonged, and remain an active participant in the child birthing [3]. If instrumental delivery is needed, EDA will help to decrease the discomfort, and if a cesarean section (CS) is required it allows to stay awake during the procedure. Moreover, using EDA helps to avoid opioid-induced maternal and neonatal respiratory depression [4]. A systematic review study, conducted in 2020, concludes that the early administration of EDA does not increase the risk of proceeding CS, prolong the second stage of labor, or impact maternal and neonatal outcomes [5]. American College of Obstetricians and Gynecologists (ACOG) recommends the use of analgesia in labor when possible [6]. The National Institute for Clinical Excellence has encouraged patients’ education on the availability and effectiveness of labor pain options, hence, ensuring that pregnant women have the choice of adequate pain management in labor [7]. EDA is proven an effective and safe option for optimal labor pain management [2,4]. Therefore, it is imperative to assess the perception and acceptability of our population to EDA in order to educate and correct misconceptions. Therefore, we conducted this study to assess pregnant women's awareness, perception, and desirability of the usage of EDA, and to identify the reasons for giving birth without epidural analgesia.

## Materials and methods

Methods

The study aims to assess the awareness, perception, and desirability of epidural analgesia among pregnant women, identify the reasons for giving birth without epidural analgesia, and the predictors for epidural analgesia requests among pregnant women at a single center in Saudi Arabia. With the approval (IRB-UGS-2020-01-291) of Imam Abdulrahman bin Faisal University IRB, a cross-sectional study was conducted at King Fahad University Hospital (KFUH) in Al Khobar, Eastern Province of Saudi Arabia using a self-administered questionnaire. The questionnaire is divided into eight sections including demographic characteristics, frequency of EDA usage and history of exposure, experience of labor, perception of EDA, awareness of EDA, awareness of complications of EDA in labor, desirability, and source and adequacy of the information. Both paper-based and electronic versions of the questionnaire, according to patients’ convenience, were offered to all pregnant women attending antenatal clinics and obstetrics’ emergency room excluding postpartum women. With the corona virus disease 2019 (COVID-19) restrictions and reduction in number of clinic visits to maintain social distancing, an average of seven women per day was expected to agree to participate in the study. Thus, 308 women are expected to participate in the two months period. The questionnaire was developed by the researchers based on a thorough literature review of prior studies [2,8,9]. It was developed in English then translated to Arabic. The English version was provided for non-Arabic patients. A pilot study for both the Arabic and English version validation was performed on 10 participants who weren’t included in the study and changes were made accordingly. The data collectors explained the study to candidates, after agreeing on participation, and signed consent was taken from all participants before they were given the self-administered questionnaire to complete.

Data analysis

The data were presented as frequency tables for categorical variables. Chi-squared test was used to compare demographics as an independent variable (Age, Nationality, Income, and Parity) with perception, awareness, and desirability of EDA as dependent variables. The questionnaire was assessed and showed that Cronbach's Alpha is 0.859 which means it is statistically reliable.

The result in terms of awareness, perception and desirability, were interpreted using the mean as follows: from 1 to 2 considered to be positive, and from 2.1 to 3 considered to be negative. Results with p-values below 0.05 were considered to be statistically significant. All data analyses were performed in IBM SPSS Statistics for Windows, Version 26.0 (Released 2019, IBM Corp, Armonk, New York).

## Results

For a two-month study period, a total of 209 pregnant women participated in this study with a participation rate of 67.5%. Some of the participants completed the questionnaire but there were also some missing data. The majority of women were between the age of 25-35 years old (62%) and predominantly Saudi (80.6%). Moreover, approximately half of the respondents' income was less than 8000 Saudi Riyal (SAR) (49.8%). The majority (80.6%) of our participants had given at least one birth in the past, and only 19.3% were nulliparous. Table 1 gives the details.

**Table 1:** Demographic characteristics SAR: Saudi Riyal

Factors	Descriptive statistics	
	Number (N)	Percentage (%)
Age		
15-19 years old	11	5.5
20-24 years old	22	11.0
25-29 years old	58	29.0
30-35 years old	66	33.0
> 35 years old	43	21.5
Nationality		
Non-Saudi	40	19.4
Saudi	166	80.6
Income		
<8000SAR	100	49.8
8000-20000SAR	72	35.8
> 20000SAR	29	14.4
Parity		
None	40	19.3
One birth	52	25.1
Two to four births	93	44.9
More than five births	22	10.6

Out of the 209 women who participated in the questionnaire, only 68 (32.7%) used epidural analgesia in their previous labor. Fifty-two participants reported a negative experience in the form of either developing side effects or complications such as inadequate analgesia and back pain (39.7% and 36.8%, respectively). On the other hand, 76.8% of women who had used epidural analgesia reported a satisfactory experience. Only 28.6% of the women who did not use epidural analgesia in the previous labor are willing to use it in the future. The reasons for rejection were mostly due to fear of side effects (40%). See Table 2.

**Table 2:** Frequency of epidural usage and history of exposure

Question	Descriptive statistics	
	Number (N)	Percentage (%)
Have you ever had epidural analgesia?		
No	140	67.3
Yes	68	32.7
Did you develop any side effects?		
No	41	60.3
Yes	27	39.7
Did you develop any complication?		
No	43	63.2
Yes	25	36.8
What was your experience from using epidural analgesia in labor?		
Satisfied	53	76.8
Not satisfied	15	21.7
Not available	1	1.4
Would you like to use epidural analgesia in future?		
No	38	27.1
Yes	40	28.6
Unsure	52	37.1
Not available	10	7.1
Reason for rejecting epidural analgesia		
Culture	2	1.4
Fear of side effects	56	40.0
I want natural	9	6.4
Expensive	4	2.9
I have no reason	38	27.1
Not available	31	22.1

Although pregnant women who already used EDA reported positive perception, awareness, and awareness of complications of EDA, the relation between previous use of EDA with the perception, awareness, and awareness of complications of EDA was statistically insignificant (p-value = 0.503, 0.616, 0.335 respectively).

Around 56% of participants were worried about losing the ability of sensation when they need to push; however, half of them have a positive perception of EDA and agreed that the benefits outweigh the risks of potential side effects. See Table 3.

**Table 3:** Perception of EDA EDA: Epidural Analgesia

Description (n=209)		Response
Percentage	Number	
The benefits of pain relief outweigh the risk of side effects with EDA		
51.2%	107	Agree
14.8%	31	Disagree
34.0%	71	Unsure
I am worried about the possibility of more complications happening with the labor if using EDA		
67.0%	140	Agree
16.3%	34	Disagree
16.7%	35	Unsure
I would NEVER have an EDA		
30.0%	62	Agree
35.7%	74	Disagree
34.3%	71	Unsure
I am worried that I might not be able to feel when I need to push		
56.5%	118	Agree
18.7%	39	Disagree
24.9%	52	Unsure
I am worried that I may lose control of my urination		
45.9%	96	Agree
24.9%	52	Disagree
29.2%	61	Unsure

The patient’s perception is significantly associated with age (p-value 0.049). In other words, the majority of women with a positive perception toward EDA were in the age group of 30-35 years (33.7%). Similar results were found between income and perception, which were statistically significant; participants with low income (<8000SAR) showed more positive perceptions regarding the use of EDA for labor pain. However, parity and nationality did not significantly affect the women's perception of EDA. See Table 4.

**Table 4:** Association between awareness of EDA, awareness of EDA complications, perception and desirability toward EDA, and socio-demographic characters EDA: Epidural Analgesia; N: Number

Variable		Awareness of EDA			Awareness of complications of EDA in labor			Perception of EDA				Desirability		
		Positive	Negative	p-value	Positive	Negative	p-value	Positive	Negative	p-value	Positive		Negative	p-value
Age														
15-19 years old	N	8	3	0.544	11	0	0.087	11	0	0.049	8		3	0.982
	%	4.7	10.7		6.8	0.0		6.3	0.0		6.3		4.3	
20-24 years old	N	20	2		18	4		20	2		14		8	
	%	11.6	7.1		11.1	10.5		11.4	8.0		10.9		11.4	
25-29 years old	N	52	6		51	7		53	5		37		20	
	%	30.2	21.4		31.5	18.4		30.3	20.0		28.9		28.6	
30-35 years old	N	55	11		52	14		59	7		41		24	
	%	32.0	39.3		32.1	36.8		33.7	28.0		32.0		34.3	
> 35 years old	N	37	6		30	13		32	11		28		15	
	%	21.5	21.4		18.5	34.2		18.3	44.0		21.9		21.4	
Nationality														
Non-Saudi	N	31	9	0.113	35	5	0.218	37	3	0.277	30		9	0.066
	%	17.6	30.0		21.1	12.5		20.6	11.5		22.9		12.3	
Saudi	N	145	21		131	35		143	23		101		64	
	%	82.4	70.0		78.9	87.5		79.4	88.5		77.1		87.7	
Income														
<8000SAR	N	83	17	0.349	88	12	0.012	90	10	0.028	54		45	0.009
	%	48.0	60.7		55.0	29.3		51.1	40.0		42.2		63.4	
8000-20000SAR	N	63	9		52	20		65	7		51		21	
	%	36.4	32.1		32.5	48.8		36.9	28.0		39.8		29.6	
> 20000SAR	N	27	2		20	9		21	8		23		5	
	%	15.6	7.1		12.5	22.0		11.9	32.0		18.0		7.0	
Parity														
None	N	36	4	0.702	32	8	0.858	35	5	0.350	21		19	0.140
	%	20.2	13.8		19.2	20.0		19.3	19.2		16.0		25.7	
One birth	N	43	9		44	8		49	3		30		20	
	%	24.2	31.0		26.3	20.0		27.1	11.5		22.9		27.0	
Two to four births	N	81	12		74	19		78	15		67		26	
	%	45.5	41.4		44.3	47.5		43.1	57.7		51.1		35.1	
more than five births	N	18	4		17	5		19	3		13		9	
	%	10.1	13.8		10.2	12.5		10.5	11.5		9.9		12.2	

Data on the awareness of epidural analgesia (Table 5) showed that 69.4% of women were aware of the technique of EDA. Unfortunately, 22% of the women thought that EDA was not available in our institution. In addition, just as the details of the technique are important, knowing the profession of the provider of EDA is important as well. More than half the women (73.8%) agreed that not any healthcare worker can provide them with EDA. In regard to the misconception that EDA may increase the risk of caesarian delivery, in this study there was a clear confusion regarding this issue as 41.1% were unsure about the answer while 31.4% disagreed. Concerning lower limb weakness and paraplegia, analysis of the result showed that most of the answers agreed with both of the misconceptions (45.5% and 42.1% respectively). EDA is used to help the women to deliver with less pain; however, 42.6% of the women agreed that contractions become weak or stop completely after the administration of EDA.

**Table 5:** Awareness of EDA EDA: Epidural Analgesia; C‑section: Cesarean Section; KFUH: King Fahad University Hospital

Description (n=209)		Response
Percentage	Number	
I am aware of EDA for labor pain		
77.5%	162	Agree
3.3%	7	Disagree
19.1%	40	Unsure
Are you aware it is provided in KFUH?		
62.2%	130	Agree
9.6%	20	Disagree
27.9%	58	Unsure
EDA is the administration of a local anesthetic through a catheter into the epidural space of the spine		
69.4%	145	Agree
3.3%	7	Disagree
27.3%	57	Unsure
EDA is one of the best forms of pain relief in labor		
68.8%	143	Agree
5.8%	12	Disagree
25.5%	53	Unsure
Any healthcare worker can perform EDA		
8.7%	18	Agree
73.8%	152	Disagree
17.5%	36	Unsure
EDA increases the probability of C‑section		
27.5%	57	Agree
31.4%	65	Disagree
41.1%	85	Unsure
EDA may cause lower limb weakness		
45.5%	95	Agree
23.9%	50	Disagree
30.6%	64	Unsure
EDA causes paraplegia		
42.1%	88	Agree
25.4%	53	Disagree
32.5%	68	Unsure
Contractions become weak or stop completely after administration		
42.6%	89	Agree
19.6%	41	Disagree
37.8%	79	Unsure
EDA reduces labor pain and allows the mother to push when needed		
57.4%	120	Agree
10.0%	21	Disagree
32.5%	68	Unsure

Awareness of EDA complications was assessed by asking about the common complication of EDA (Table 6). Most of the women (n=120; 57.4%) agreed that it causes back pain followed by headache (n=96; 45.9%), low blood pressure (n=77; 36.8%), increased intervention in labor (n=63; 30.1%), and finally prolongation of second-stage labor and effect on the unborn baby (n=61; 29.2%). There was positive awareness of EDA complications, which was associated with income. The result revealed that 55% of participants’ income was less than 8000SAR. The p-value result from studying the association between income and awareness of complications of EDA was found to be significant at 0.012. On the other hand, data showed that there is no significant association between age, nationality, parity, and awareness of EDA complications (Table 4).

**Table 6:** Awareness of EDA complications EDA: Epidural Analgesia

Description (n=209)		Response
Percentage	Number	
EDA cause back pain		
57.4%	120	Agree
14.4%	30	Disagree
28.2%	59	Unsure
EDA cause headache		
45.9%	96	Agree
15.8%	33	Disagree
38.3%	80	Unsure
EDA increased intervention in labor		
30.1%	63	Agree
19.6%	41	Disagree
50.2%	105	Unsure
EDA cause low blood pressure		
36.8%	77	Agree
15.3%	32	Disagree
47.8%	100	Unsure
EDA cause prolongation of second-stage labor and effect on the unborn baby		
29.2%	61	Agree
21.1%	44	Disagree
49.8%	104	Unsure

Regarding desirability of EDA, the majority of participants would have EDA in certain circumstances, for example, if they got fatigued or when recommended by a midwife or physician. Furthermore, approximately half of the responders (51.2%) would have EDA as their first choice for pain relief.

In this study result, there was a strong association between income and desirability to take EDA. As shown in Table 4, pregnant women with income less than 8000SAR (p-value 0.009) were more willing to use EDA than the others. On the other hand, there was no significant association between age, nationality, parity, and desirability to use EDA. The end result revealed that most of the pregnant women in this study (63.6%) had positive desirability to take EDA.

Most women (n=71; 34%) reported family and friends as their source of information about EDA, followed by doctors (n=60; 28.7%), media (n=56; 26.8%), nurses (n=9; 4.3%), and antenatal class (n=9; 4.3%).

In addition, pregnant women who received information from the antenatal class have better perception and awareness of EDA compared to those receiving information from other sources (100%, 88.9% respectively). However, this relation was statistically insignificant (p-value 0.125, 0.947 respectively).

## Discussion

Our study showed that the majority of participants (85.6%) were aware of the EDA. A similar result was found in a study done in Riyadh, which showed that the participants were knowledgeable regarding EDA [10]. In contrast, a study done in Abu Dhabi in 2015 showed that women are not well informed about EDA and so they do not consider it [11]. Moreover, there was no relation found between the awareness of EDA and age, nationality, income, and parity (p-value 0.544, 0.113, 0.349, 0.702 respectively).

Of the women in our study population, 80.4% were aware about the complication of using EDA for labor pain. This was contrary to study findings by Ezeonu et al., which showed that majority of women (72.41%) had no knowledge about the complication of EDA [2]. We found that the majority of women agreed that EDA causes back pain and headache. This finding is similar to what was found in a Saudi study that was done in 2018 [12], unlike the conclusion of another study that was done among women from Australia and the United Kingdom, where they found that women in Australia have greater awareness about epidural infection and nerve damage [13].

The overall perception regarding EDA was positive in 87.6% of participants, which is higher by approximately 37% than the result of a Saudi study conducted in 2018 [12]. This could indicate that the population is becoming more educated toward EDA. A study in Townsville, Australia, showed that 44% of participants disagreed that they would never use EDA, while 56% were unsure about whether EDA helps with labor progression, and 63% agreed that they were worried about the possibility that it will affect their ability to walk and move around [8]. These results were similar to our study with a small difference in the percentages (Table 3). In two previous studies, it was seen that most of the participants in Saudi Arabia and Pakistan were concerned about backache associated with EDA [14,12]. Similarly, in this study, back pain if using EDA was the biggest concern.

When it comes to EDA desirability, only 63.6% of pregnant women showed willingness to take EDA. In a study done in ﻿Ludhiana, desirability and willingness to take EDA (96.7%) were significantly higher than our findings [2]. Interestingly, we found that pregnant women with income less than 8000SAR (p-value 0.009) were more willing to use EDA. We also found that age, nationality, and parity did not affect desirability to have EDA in labor. In contrast, a previously published study in India found that unwillingness and desire for epidural analgesia were closely linked to the gravida status [15]. 

Logically, we found that pregnant women who had a satisfactory experience with EDA (36, 67.9%) are more willing to use it again compared to those with unsatisfactory experience (p-value 0.000). However, only 28.6% of pregnant women who had not used EDA in the past would like to use it in future. In addition, the reasons for low desirability for EDA were due to fear of side effects. This end result is similar to another study done in Nigeria, which concluded that 95% of the respondents who took EDA were satisfied with the experience and desired to take it again [2]. However, only 70% of those who had not used EDA expressed the desire to use it. Some of the reported causes for the lack of desirability to using EDA includes fear of side effects, cost, and experiencing natural labor.

The most common source of information regarding EDA was the family and friends (34%) followed by physicians (28.7%), media (26.8%), nurses (4.3%), and antenatal class (4.3%). This result is similar to a study done in Saudi Arabia where they found that the most common source of information regarding EDA was friends and relatives (67%), previous labor experience (16%), media (8%), and books (2%) respectively [12]. Only 39.1% of participants agreed that they have received adequate information on various forms of pain relief in labor including EDA. Therefore, antenatal care programs should include educational sessions about different types of pain relief during labor including EDA. 

Application

Using the result of this research, an antenatal care program should include a discussion between pregnant women and a certified healthcare provider about EDA, its side effects, potential complications and alternatives.

Limitations and recommendations

As the study was conducted between November and December 2020, the number of patients was restricted due to the COVID-19 pandemic. Moreover, this study took place in a tertiary hospital where the cases were more complicated; therefore, the participants were more knowledgeable. A suggestion is to conduct further research among pregnant women from different levels of healthcare systems including primary, secondary, and tertiary health care facilities.

In this study, most pregnant women received their sources of information from family, friends, or social media. This could influence the awareness, perception, and desirability of EDA. Therefore, we recommend giving antenatal educational programs for pregnant women who attend the clinic about possible pain management in labor including EDA. In addition, healthcare providers should put more effort on social media and provide the community with evidence-based information about possible methods of pain relief during labor since social media has a great influence in the community these days.

## Conclusions

This study highlights the importance of labor pain management policies and patients’ education. Although our study results demonstrate that the majority of pregnant women were aware of EDA and also had a positive perception and desirability toward using EDA for labor pain, there is still a good number of women who have a lack of awareness and perception toward EDA. These women may have more intense labor pain that will negatively affect their labor experience and make it less enjoyable. Therefore, having a national policy and guidelines on labor pain management is crucial to improving labor experience and maternal care in Saudi Arabia. Furthermore, education about different types of labor pain analgesia, especially EDA, should be included early in an antenatal care program.
